# Knockdown circTRIM28 enhances tamoxifen sensitivity via the miR-409-3p/HMGA2 axis in breast cancer

**DOI:** 10.1186/s12958-022-01011-3

**Published:** 2022-09-30

**Authors:** Shiyong Yang, Changwu Zou, Yuxin Li, Xianguo Yang, Wei Liu, Guannan Zhang, Nina Lu

**Affiliations:** Department of Oncology, Men No. 2 People’s Hospital, N0. 39, Xiangshan Road, Jingmen City, Hubei Province 448000 PR China

**Keywords:** Breast cancer, circTRIM28, miR-409-3p, HMGA2, Tamoxifen

## Abstract

**Background:**

Tamoxifen (TAM) is a frequently-used treatment for breast cancer (BC). But the TAM resistance seriously affects the patient therapeutic effect. Previous research indicated that circular RNAs (circRNAs) might participate in the regulatory processes of BC. Here, we discovered the parts of circular RNA tripartite motif-containing 28 (circTRIM28) in BC.

**Methods:**

CircTRIM28, microRNA-409-3p (miR-409-3p), and high mobility group AT-hook 2 (HMGA2) levels were perceived by qRT-PCR and western blot. Moreover, the biological functions of the cells were examined. Furthermore, dual-luciferase report was employed to reconnoiter the targeted relationship between miR-409-3p and circTRIM28 or HMGA2.

**Results:**

CircTRIM28 and HMGA2 were augmented, and the miR-409-3p was repressed in BC. Silencing circTRIM28 enhanced tamoxifen sensitivity and cell apoptosis, whereas hampered cell development in BC cells. In mechanism, circTRIM28 could sponge miR-409-3p to increase HMGA2. In addition, silencing circTRIM28 impeded tumor growth.

**Conclusion:**

CircTRIM28 facilitated the BC via miR-409-3p/HMGA2.

## Highlights

1. The expression of circTRIM28 was upregulated in BC.

2. Silencing circTRIM28 enhanced tamoxifen sensitivity and cell apoptosis, whereas curbed cell proliferation, cell migration and invasion in BC cells.

3. CircTRIM28 expedited BC by targeting miR-409-3p.

4. MiR-409-3p repressed BC by retarding HMGA2.

## Introduction

Breast cancer (BC) is common cancer worldwide. Hormone receptor (HR) positive patients accounted for three-quarters of all BC patients [1]. In the current clinical treatment, tamoxifen (TAM) is an important treatment for patients with HR-positive, especially for premenopausal patients [2, 3]. However, patients who develop resistance to TAM can make treatment difficult [4, 5]. Therefore, we urgently need to determine the drug resistance mechanism of TAM.

Circular RNAs (circRNAs) are a kind of non-coding RNAs that employ a key role in numerous illnesses [6, 7]. Hsa_circ_001783 regulates cell proliferation and invasion in BC [8]. Circ-ABCB10 promotes BC proliferation by regulating miR-1271 [9]. Besides, circ-DNMT1 activates autophagy in BC [10]. In addition, circ_103809 regulates the PI3K/AKT signaling pathway in BC progression [11]. However, the definite monitoring mode of circTRIM28 in BC is not clear.

MicroRNAs (miRNAs) are a class of RNAs that affects the cellulate processes [12, 13]. MiR-409-3p inhibits the evolution of tongue squamous cell carcinoma [14]. Besides, miR-409-3p participates in regulating the cell proliferation of osteosarcoma [15]. Moreover, miR-4732-5p regulates BC progression via TSPAN13 [16]. CircDENND4C regulates glycolysis in BC [17]. All the same, the identification of miR-409-3p in BC was not yet testified. Previous research has reported that the high mobility group AT-hook 2 (HMGA2) is connected with many cancer developments, like differentiation and angiogenesis. HMGA2 has been shown to be an effective carcinogen [18]. Yet, the influence of HMGA2 in BC cells is blurry.

Up to date, increasing attention has been paid to the competing endogenous RNA (ceRNA) mechanism, which proposed that endogenous RNA transcripts with microRNA (miRNA) response elements (MREs) might derepress target mRNAs expression via sponging miRNA [19, 20]. Of note, the mechanism has been considered the major regulation manner for circRNAs [21, 22]. Herein, the online bioinformatics tools exhibited that there were some binding sites between circTRIM28 and miR-409-3p for the first time. Therefore, our purpose is to analyze the function of circTRIM28/miR-409-3p in BC and reconnoiter possible governing network basics on these effects.

## Materials and methods

### Clinical tissues

The test was approved by Jing Men No. 2 People’s Hospital. Sixty-four BC tissues (BC-S: 30 cases were sensitive to tamoxifen; BC-R: 34 cases were resistant to tamoxifen) and normal tissue (*n* = 64) were collected from Jing Men No. 2 People’s Hospital. All volunteers wrote the informed consent. Besides, the clinicopathological features of these patients were shown in  Table 1.

**Table 1:** Relationship between circTRIM28 expression and clinicopathologic features of BC patients

	Characteristics *n* = 64	circTRIM28 expression		*P* value
		Low (*n* = 32)	High (*n* = 32)	
Age (years)				0.8013
≤ 50	28	15	13	
> 50	36	17	19	
TNM grade				0.0023*
I + II	29	21	8	
III	35	11	24	
Lymph node metastasis				0.0047*
Positive	38	13	25	
Negative	26	19	7	
Tumor size				0.0003*
≤ 2 cm	25	20	5	
> 2 cm	39	12	27	

*TNM* tumor-node-metas-tasis; *P < 0.05

### Cell lines

The BC cell lines MCF7 and MDA-MB-231 with MCF10A as control. MCF7 cells a human breast cancer cell line with estrogen, progesterone and glucocorticoid receptors. When grown in vitro, the cells are capable of forming domes and the epithelial-like cells grow in monolayers. MDA-MB-231 cells were chosen as an ER negative cell line with invasive phenotype in vitro, and have epithelial-like morphology and appears phenotypically as spindle-shaped cells. All cells were purchased from the American type culture collection (ATCC, Manassas, VA, USA) and used at between three and ten passages. Cells were grown in RPMI 1640 supplemented with 10% fetal bovine serum (FBS) and penicillin/streptomycin at 37 °C in a 5% CO2 and 95% humidified atmosphere at a frequency of 2–3 times/week. According to the https://web.expasy.org/cellosaurus/CVCL_0023 website, the cells have no cross-infection. MCF7 and MDA-MB-231 cells were treated with 0.1 μM 4-hydroxytamoxifen (4-OH-TAM) (Sigma, St. Louis, MO, USA) for at least 6 months to produce anti-TAM BC cells, named MCF7/R and MDA-MB-231/R. The TAM-resistant phenotype of BC cells was maintained using the same medium supplemented with 0.5 μM TAM.

### QRT-PCR

Whole RNAs were utilized by Trizol reagent (Takara, Tokyo, Japan) and reverse transcription as cDNA. Afterward, the cDNA level was quantified by SYBR Green kit (Takara). Successively, the data were assessed by the 2^-△△Ct^ method and standardized by U6 or GADPH. Primers were listed in Table 2.

**Table 2:** Primers for PCR

Name		Primers for PCR (5′-3′)
circTRIM28	Forward	CAGGAGAAGTTGTCACCTCCC
	Reverse	ACTTGGCTGGCCCTCAGTTA
HMGA2	Forward	CAGCAAGAACCAACCGGTGA
	Reverse	GGATGTCTCTTCAGTTTCCTCCT
miR-409-3p	Forward	GTATGAGAATGTTGCTCGGTGA
	Reverse	CTCAACTGGTGTCGTGGAG
miR-217	Forward	GTATGATACTGCATCAGGAACTG
	Reverse	CTCAACTGGTGTCGTGGAG
miR-524-3p	Forward	GCGGAGGAAGGCGCTTCCCTT
	Reverse	CTCAACTGGTGTCGTGGAG
miR-525-3p	Forward	GTATGAGAAGGCGCTTCCCTT
	Reverse	CTCAACTGGTGTCGTGGAG
U6	Forward	CTCGCTTCGGCAGCACATATACT
	Reverse	ACGCTTCACGAATTTGCGTGTC
GAPDH	Forward	TCCCATCACCATCTTCCAGG
	Reverse	GATGACCCTTTTGGCTCCC

### Western blot

The method described by Hou et al. [23]. The antibodies were listed: anti- HMGA2 (ab207301; 1:1000 dilutions; 0.693 μg/mL; Abcam, Cambridge, MA, USA), anti-PCNA (ab92552; 1:1000 dilutions; 0.164 μg/mL; Abcam), anti-Cleaved-caspase 3 (ab32042; 1:500 dilutions; 3.088 μg/mL; Abcam), anti-MMP9 (ab76003; 1:10,000 dilutions; 0.186 μg/mL; Abcam), and anti-β-actin (ab8227; 1:1000 dilutions; 0.1 μg/mL; Abcam).

### Cell transfection

The sh-circTRIM28 (sh-circTRIM28#1, sh-circTRIM28#2, sh-circTRIM28#3), the control (sh-NC), miR-409-3p mimics, miR-409-3p inhibitors (anti-miR-409-3p) and controls, Bio-NC, Bio-miR-409-3p, Oligo probe, circTRIM28 probe, HMGA2 overexpression (HMGA2) and control plasmid (vector) were also fabricated by Ribobio (Guangzhou, China). Lipofectamine 2000 (Sigma) was employed in transfection.

### RNase R and act D assay

The circ_0047921 and CD226 mRNA were treated with RNase R (Sigma). Similarly, actinomycin D (Act D, 2 mg/mL) or DMSO (Sigma) was added to the medium as control, and the tests were performed respectively. Finally, the expression level of circ_0047921 and CD226 mRNA were uncovered by qRT-PCR.

### CCK8 assay and cytotoxicity assay

After post-transfection, BC cells (2.0 × 10^3^/well) were seeded in 96-well plates. The cells in each well were exposed to different doses of tamoxifen (0, 5, 10, 20, 30 or 40 μM) for 48 h. Next, CCK8 (Sigma) was supplemented and the half-maximal inhibitory concentration (IC_50_) with assessed. A similar model was enforced to measure cell viability.

### Cell proliferation assay

BC cells were seeded into 96-well plates. Then, the EdU Apollo In Vitro Imaging Kit (Sigma) was employed along with the guide. Generally, tumor cells were incubated with 50 μM EdU for 2 h at 37 °C and fixed in 4% formaldehyde. After being stained with Apollo reaction cocktail and DAPI (identify the nuclei) for 30 min, the proliferation-positive cells were photographed and counted under a microscope.

### Colony formation assay

In short, 1 × 10^3^ BC cells were plated into 6-well plates and maintained for 2 weeks. Finally, the colonies were fixed with methanol for 20 min and dyed with 0.1% crystal violet (Sigma) at room temperature. Finally, cell colonies were counted and photographed.

### Flow Cytometry assay

BC cells with transfection were seeded in 6-well plates. The apoptotic was assessed by.

Annexin V-FITC/PI kit (Sigma) with a flow cytometer. In short, tumor cells were trypsinized and washed three times in PBS, followed by re-suspending in binding buffer. After being dual-stained with 5 μL Annexin V-FITC and 10 μL PI solution in the dark for 10 min, the apoptotic cells were identified according to a flow cytometer.

### Transwell assay

BC cells were plated into a transwell with a pore polycarbonate membrane (BD Bio-sciences, Bedford, MA, USA). In brief, 4 × 10^5^ transfected BC cells were planted into the top chamber. Then the lower chamber of the transwell contains 500 μL of DMEM and 10% FBS. Succeeding, the inferior surface of the membrane cells was stained. The same method was enforced to detect the invasion ability, but the transwell chamber was precoated with matrigel (BD Biosciences). Eventually, a light microscope was performed to validate the count of cells.

### Dual-luciferase reporter assay

The targeted combination relationship of miR-409-3p and circTRIM28 or HMGA2 was predicted by circbank (http://www.circbank.cn/), starbase (http://starbase.sysu.edu.cn), and circinteractome (https://circinteractome.nia.nih.gov). Then, the circTRIM28 and HMGA2 wild and mutant were synthesized by Ribobio (circTRIM28-WT, HMGA2–3’UTR-WT or circTRIM28-MUT, HMGA2–3’UTR-MUT). Finally, luciferase activity was quantified.

### RNA pull-down

Bio-miR-409-3p and Bio-NC were synthesized from RiboBio. The RNA pull-down assay was applied as beforehand reported [24]. Finally, circTRIM28 and miR-409-3p contents were assessed.

### RIP assay

The Magna RNA immunoprecipitation kit (Millipore, Billerica, MA, United States) was enforced to carry out RIP assay in accordance with the guide. To be brief, tumor cells at 80% confluency were harvested, followed by lysing in complete RIP lysis buffer. Then, the obtained cell lysates were incubated with anti-AGO2 or anti-IgG for 4 h at 4 °C before treating magnetic protein A/G beads for 2 h. Subsequently, the beads were washed, and the extracted total RNA was subjected to qRT-PCR analysis.

### In vivo assay

All-female BALB/C nude mice (6-week-old, 18–22 g) were bought from Shanghai Laboratory Animal Company (SLAC, Shanghai, China) under a pathogen-free environment at a temperature of 25 °C and relative air humidity between 45 and 50% with a 12/12 h day/night cycle. The in vivo work was approved by the guidance of the Animal Care and Use Committee of Jing Men No. 2 People’s Hospital. MDA-MB-231/R cells (1 × 10^6^) with or without tamoxifen (5 mg/kg) transfected with the circTRIM28 knockdown vector (sh-circTRIM28) or sh-NC were vaccinated into mice (*n* = 6/group) under a specific-pathogen-free environment. The volume (mm^3^) = length×width^2^/2. Mice were sacrificed on day 35, the tumors were used for deeper level research.

### IHC assay

In short, formalin-fixed and paraffin-embedded mice tumor tissue specimens were cut into 4 μm sections, followed by staining with hematoxylin and eosin for histopathology under a microscope. To analyze cell proliferation, the Ki67 (ab92742; 1:1000 dilutions; Abcam) contents in the tumor were detected by IHC assay. The unambiguous assessment scheme as per the description of Ma et al. [25]. Ultimately, the slides were photographed.

### Statistical assay

These statistics were assembled from no less than 3 groups of repeats. The Student’s *t*-test or ANOVA was enforced to measure the difference in GraphPad Prism 7. *P*-value < 0.05 was significant.

## Results

### The circTRIM28 was augmented in BC

**Fig. 1:**
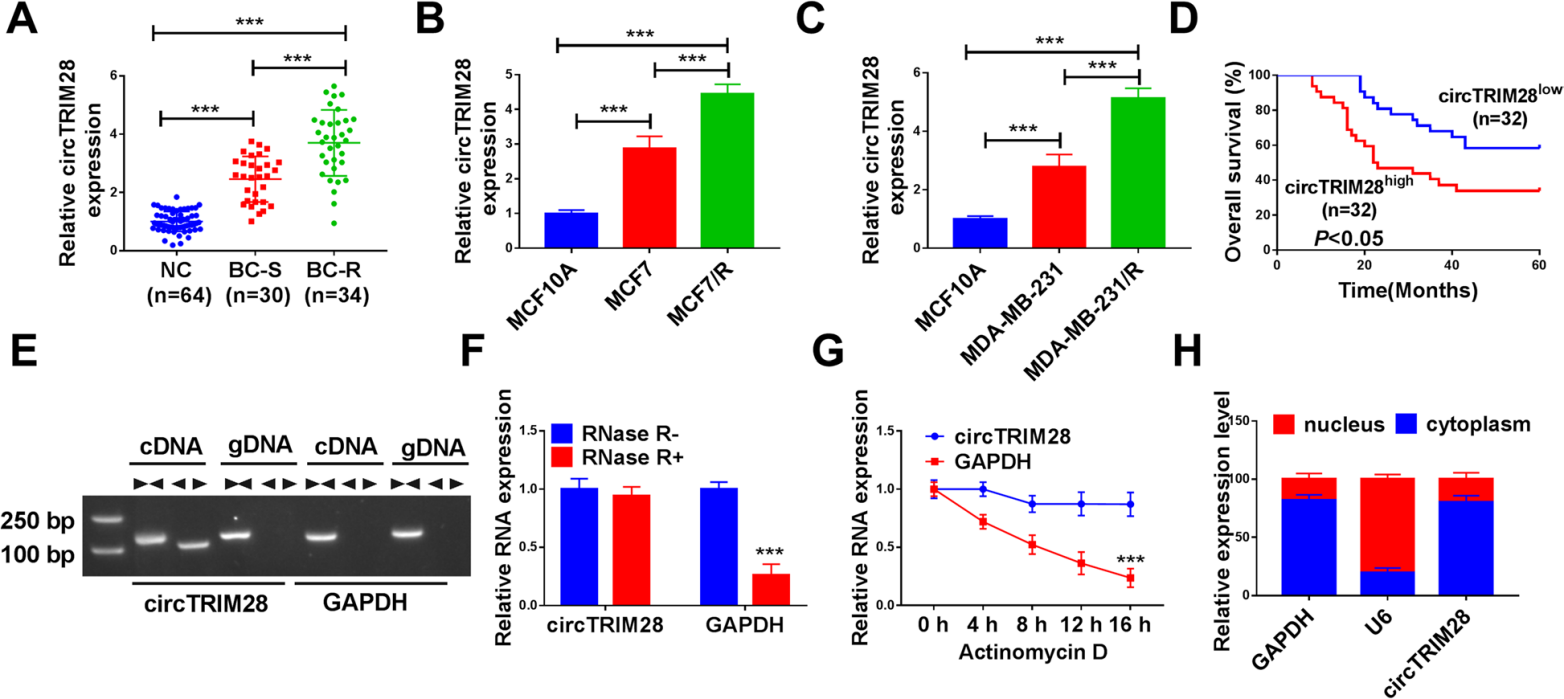
CircTRIM28 was intensified in BC. **A**-**C** The circTRIM28 level in BC. **D** The overall survival rate was measured. **E**-**G** The relative levels of circTRIM28 were detected. **H** The expression of circTRIM28 was quantified. ***P < 0.001

Hence, to further explore the role of circTRIM28 in BC, qRT-PCR was applied. CircTRIM28 was amplified in BC tissues. Besides, the expression of circTRIM28 was greatly increased in tamoxifen-resistance tissues (BC-R, *n* = 34) relative to tamoxifen-sensitive tissues (BC-S, *n* = 30) (Fig. 1A). Furthermore, our information proposed that circTRIM28 was augmented in BC cell lines (MCF7 and MDA-MB-231) than in the control cell line (MCF10A). In addition, circTRIM28 was significantly upregulated in tamoxifen-resistant BC cell lines (MCF7/R and MDA-MB-231/R) in comparison to the parental cell lines (MCF7 and MDA-MB-231) (Fig. 1B and C). Meanwhile, patients with higher circTRIM28 expression levels had significantly lower post-operative survival compared to those with lower circTRIM28 expression levels (Fig. 1D). RT-PCR usually uses two pairs of primers to exclude gene recombination, one pair of divergent primers to detect circRNA and recombinant genes, and the other pair of convergent primers to detect mRNA and circRNA [26]. Figure 1E showed that cDNA and genomic DNA (gDNA) were used as templates for detection. When cDNA was used as the template, both pairs of primers had products; when gDNA was used as the template, only convergence primers had products, indicating that the detected RNA was not produced by gene recombination and the structure pattern was circular RNA. Meanwhile, the GAPDH structure pattern was linear RNA. To further confirm the structure of circTRIM28, the RNase R enzyme and Act D treatment assay were applied to detect the structure of circTRIM28. As shown in Fig. 1F and G, the circTRIM28 was resistant to RNase R and Act D treatments, while GAPDH was impeded. In addition, the expression of circTRIM28 in the nucleus was higher than that in the cytoplasm (Fig. 1H). These outcomes exposed the circTRIM28 was upregulated in BC, and is more significantly upregulated in tamoxifen-resistant tissues and cells, which also could take effect in tamoxifen-resistant BC. Additionally, circTRIM28 structure pattern was manifested in circular RNA.

### Silencing circTRIM28 enhanced tamoxifen sensitivity and cell apoptosis, whereas inhibited cell development in BC cells

**Fig. 2:**
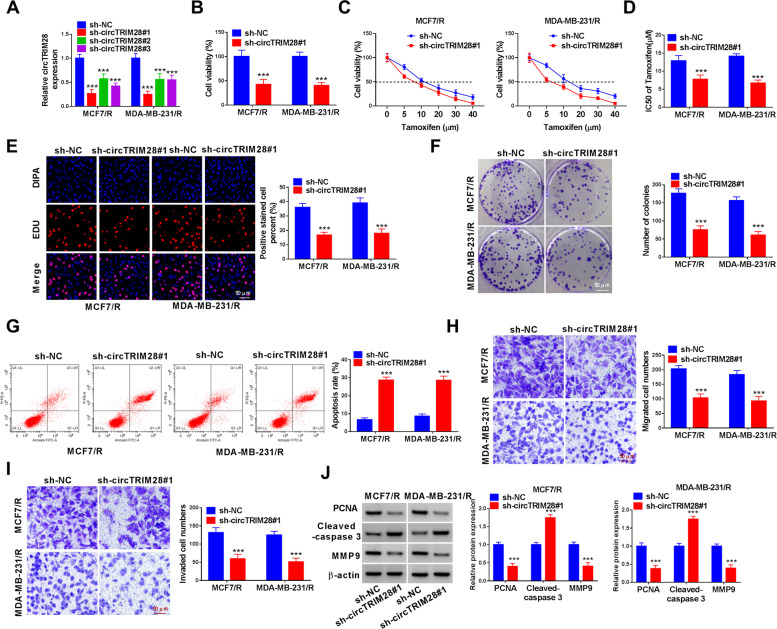
CircTRIM28 deficiency impeded BC. **A** The contents of sh-circTRIM28 were validated. **B**-**D** The cell viability and the IC50 of tamoxifen were detected. **E** and **F** The cell proliferation was examined. **G** The apoptosis of BC cells was measured. **H** and **I** The cell migration and invasion were assessed. **J** The PCNA, Cleaved-caspase 3 and MMP9 were measured. ***P < 0.001

MCF7/R and MDA-MB-231/R cells were transfected with sh-circTRIM28 (sh-circTRIM28#1, sh-circTRIM28#2, sh-circTRIM28#3), with sh-NC as control. The results designated that circTRIM28 was constrained in MCF7/R and MDA-MB-231/R cells by sh-circTRIM28 (Fig. 2A). Among them, sh-circTRIM28#1 had the most obvious inhibiting effect, so it was selected for subsequent tests. Functionally, the downregulated circTRIM28 diminished the cell vitality (Fig. 2B). In addition, the results showed that the knockdown of circTRIM28 decreased the IC_50_ value of tamoxifen (Fig. 2C and D). Besides, the EdU assay confirmed that sh-circTRIM28#1 transfection diminished the cell proliferation (Fig. 2E). Furthermore, downregulation of circTRIM28 constrained the number of colonies (Fig. 2F). Additionally, silencing circTRIM28 prompted cell apoptosis in MCF7/R and MDA-MB-231/R cells (Fig. 2G). Subsequently, the circTRIM28 deficiency inhibited invasion and migration of MCF7/R and MDA-MB-231/R cells (Fig. 2H and I). The PCNA, Cleaved-caspase 3, and MMP9 are related to cell proliferation, apoptosis, and invasion. The knockdown of circTRIM28 significantly repressed the level of PCNA and MMP9, whereas increased the expression of Cleaved-caspase 3 (Fig. 2J). Our results indicated that circTRIM28 deficiency enhanced tamoxifen sensitivity and cell apoptosis, but repressed cell proliferation, cell migration and invasion in BC cells.

### MiR-409-3p targeted circTRIM28

**Fig. 3:**
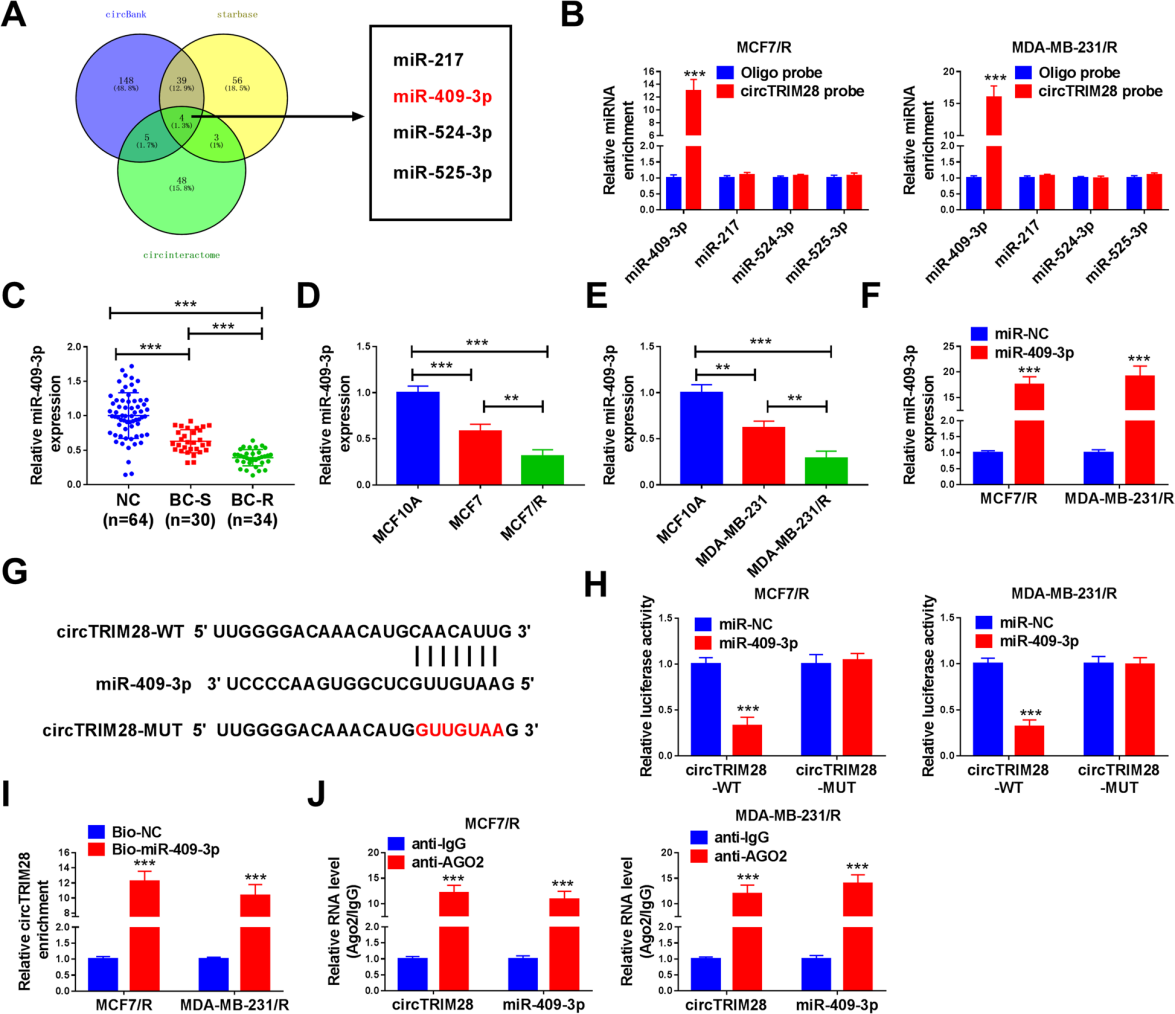
CircTRIM28 targeted miR-409-3p. **A** The binding miRNAs of circTRIM28 were predicted by circBank, starbase and circinteractome. **B** The miR-409-3p, miR-217, miR-524-3p, and miR-525-3p levels were quantified. **C**-**F** The miR-409-3p contents in BC tissues and cells. **G** The target sequence between circTRIM28 and miR-409-3p. **H**-**J** Dual-luciferase reporter assay, RNA pull-down assay, and RIP assay were employed to prove the connection of circTRIM28 and miR-409-3p. **P < 0.01, ***P < 0.001

CircBank, starbase and circinteractome was enforced to foresee the target miRNA of circTRIM28. Among them, four miRNAs overlapped, namely miR-217, miR-409-3p, miR-524-3p, and miR-525-3p (Fig. 3A). Furthermore, we revealed that miR-409-3p was amplified by circTRIM28 probe, but there were no significant changes in other miRNAs (Fig. 3B). Therefore, miR-409-3p was used as the target miRNA for subsequent tests. Besides, the miR-409-3p level in BC tissues (*n* = 64) was significantly decreased than tumor-adjacent normal tissues (*n* = 64). Meanwhile, miR-409-3p was greatly diminished in tamoxifen-resistance tissues (BC-R, *n* = 34) relative to tamoxifen-sensitive tissues (BC-S, *n* = 30) (Fig. 3C). Moreover, our numbers also displayed that miR-409-3p was downregulated in BC cell lines (MCF7 and MDA-MB-231) relative to the control cell line (MCF10A). In addition, miR-409-3p was retarded in tamoxifen-resistant BC cell lines (MCF7/R and MDA-MB-231/R) in comparison to the parental cell lines (MCF7 and MDA-MB-231) (Fig. 3D and E). Furthermore, miR-409-3p was amplified by miR-409-3p mimics (Fig. 3F). Figure 3G showed the targeted binding site of miR-409-3p and circTRIM28. Results showed that the luciferase activity was lessened in circTRIM28-WT and that miR-409-3p co-transfected in MCF7/R and MDA-MB-231/R cells versus miR-NC groups even though no alteration was observed between circTRIM28-MUT co-transfection groups (Fig. 3H). The RIP and RNA pull-down assay confirmed the targeted relationship of miR-409-3p and circTRIM28 (Fig. 3I and J).

### CircTRIM28 expedited BC by binding miR-409-3p

**Fig. 4:**
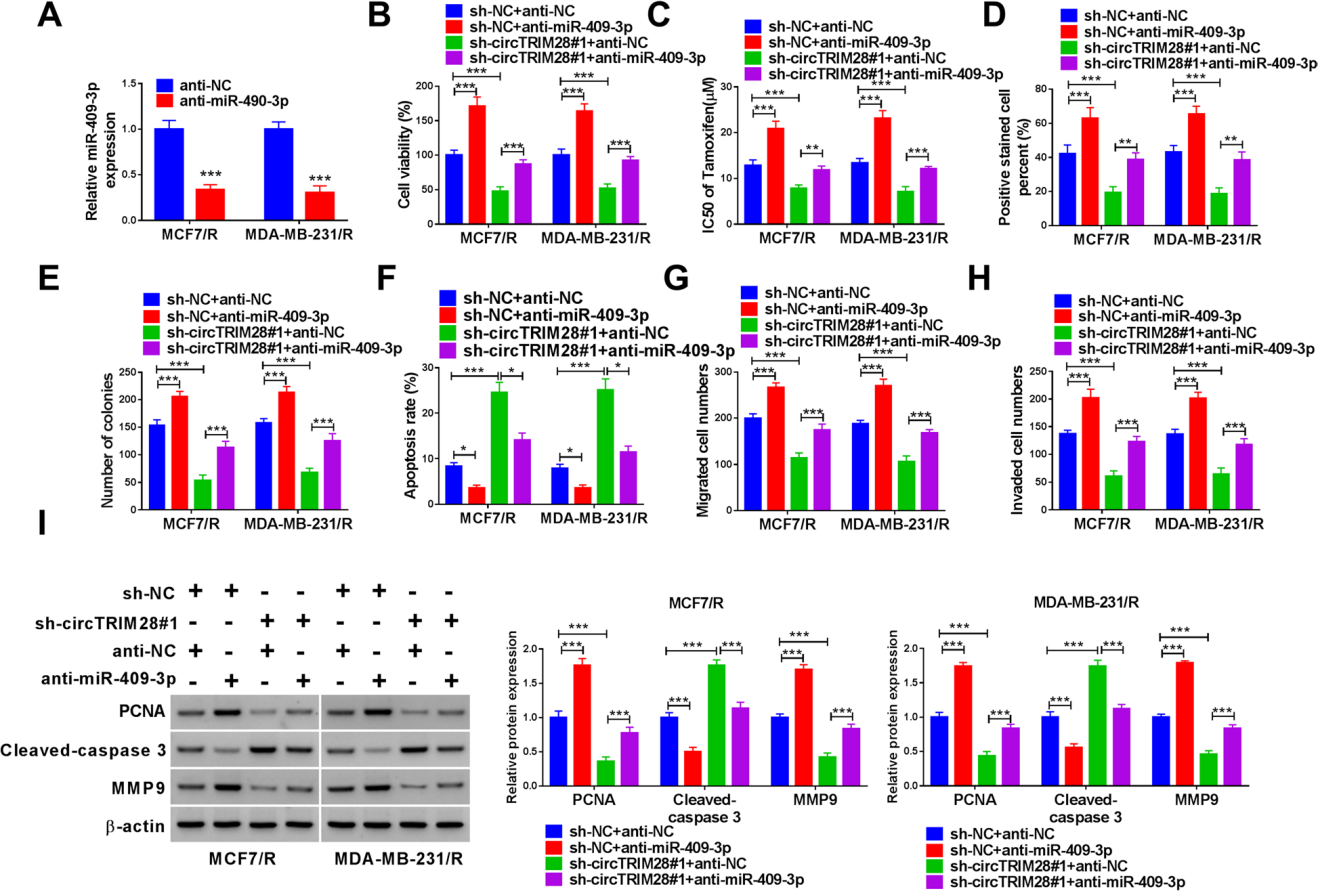
CircTRIM28 expedited BC via miR-409-3p. **A** MiR-409-3p level was quantified. **B** The cell viability, **C** the IC50 of tamoxifen, **D** the cell proliferation, **E** the number of colonies, **F** the rate of apoptosis, **G** and **H** the cell migration and invasion, **I** the protein level of PCNA, Cleaved-caspase 3 and MMP9 were measured. *P < 0.05, **P < 0.01, ***P < 0.001

MiR-409-3p content was limited in MCF7/R and MDA-MB-231/R cells by anti-miR-409-3p (Fig. 4A). MCF7/R and MDA-MB-231/R cells were transfected with sh-NC + anti-miR-409-3p and sh-circTRIM28#1 + anti-miR-409-3p, with sh-NC + anti-NC and sh-circTRIM28#1 + anti-NC as the control. We revealed that knockdown miR-409-3p could enhance the cell vitality and IC_50_ value of tamoxifen, and reverted the effect of sh-circTRIM28#1 (Fig. 4B and C). For further research, we found that anti-miR-409-3p promoted cell proliferation and reverted the effect of silencing circTRIM28 (Fig. 4D and E). Additionally, the anti-miR-409-3p hindered the cell apoptosis, and reverted the effect of sh-circTRIM28#1 (Fig. 4F). Besides, we detected that anti-miR-409-3p promoted cell invasion and migration and reverted the effect of silencing circTRIM28 (Fig. 4G and H). Afterward, the anti-miR-409-3p could increase the level of PCNA and MMP9 and diminished the Cleaved-caspase 3 level, and reverted the effect of sh-circTRIM28#1 (Fig. 4I).

### MiR-409-3p bound to HMGA2 in MCF7/R and MDA-MB-231/R cells

**Fig. 5:**
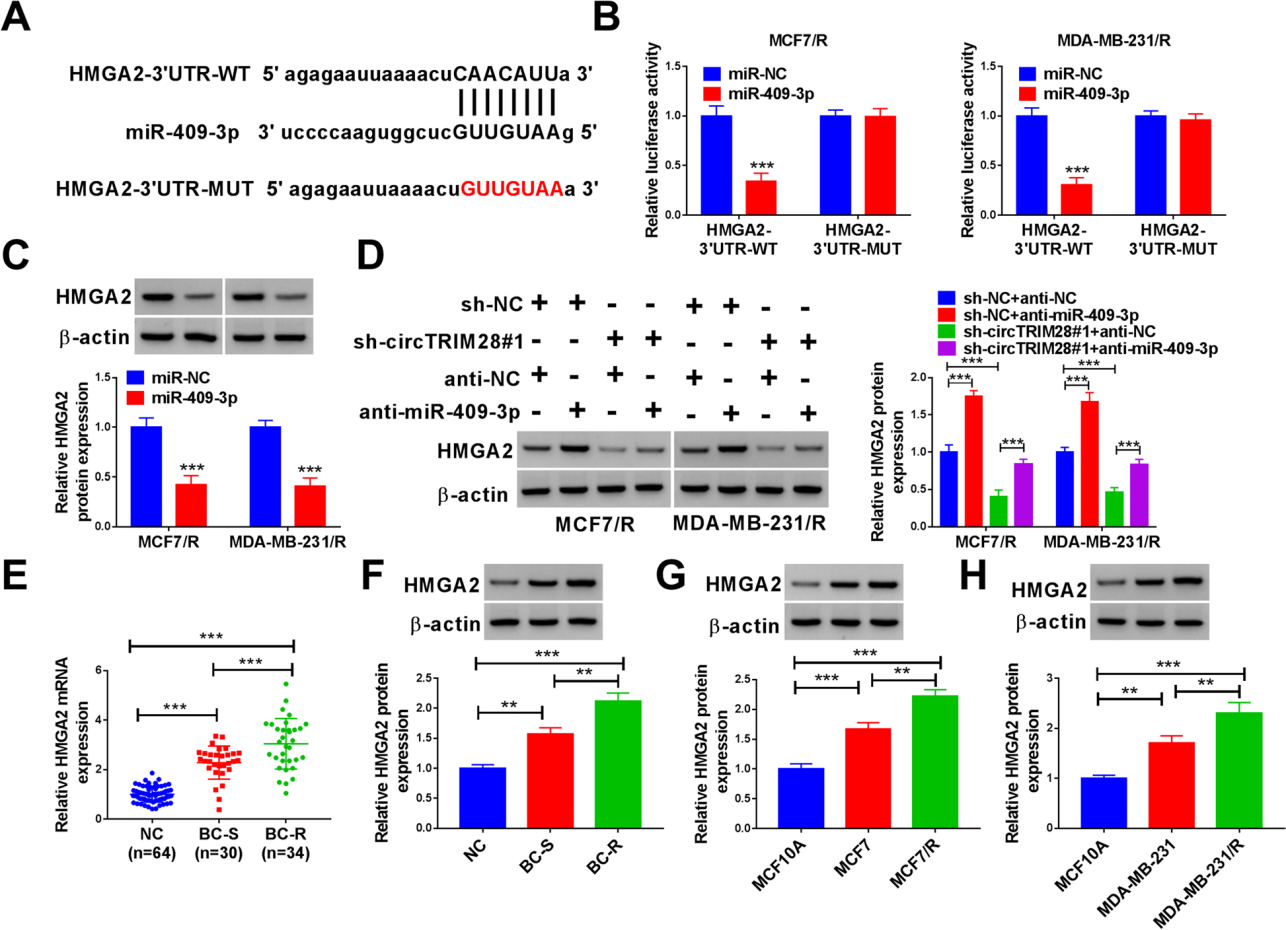
MiR-409-3p targeted HMGA2 in BC cells. **A** The targeted site between miR-409-3p and HMGA2. **B** and **C** The connection between miR-409-3p and HMGA2 was detected. **D**-**H** The HMGA2 contents were detected. **P < 0.01, ***P < 0.001

The bound sites of miR-409-3p in HMGA2 3’UTR were enforced by StarBase (Fig. 5A). Luciferase activity declined when BC cells with miR-409-3p mimics and HMGA2–3’UTR-WT co-transfected. But the luciferase activity of BC cells that contained the HMGA2–3’UTR-MUT was not altered by miR-409-3p (Fig. 5B). Additionally, HMGA2 was notably decreased by miR-409-3p (Fig. 5C). Furthermore, western blot assay was performed to illustrate that HMGA2 was amplified by anti-miR-409-3p, whereas decreased by sh-circTRIM28#1. The anti-miR-409-3p reverted the effect of sh-circTRIM28#1 on the levels of HMGA2 (Fig. 5D). Besides, HMGA2 in BC tissues (*n* = 64) was upregulated compared with tumor-adjacent normal tissues (*n* = 64). Meanwhile, the expression of HMGA2 was greatly increased in tamoxifen-resistance tissues (BC-R, *n* = 34) relative to tamoxifen-sensitive tissues (BC-S, *n* = 30) (Fig. 5E and F). What is more, our information demonstrated that HMGA2 was enhanced in BC cell lines (MCF7 and MDA-MB-231) than MCF10A cells. In addition, HMGA2 was significantly higher in tamoxifen-resistant BC cell lines (MCF7/R and MDA-MB-231/R) in comparison to the parental cell lines (MCF7 and MDA-MB-231) (Fig. 5G and H).

### CircTRIM28 facilitated the progression of BC by regulating HMGA2

**Fig. 6:**
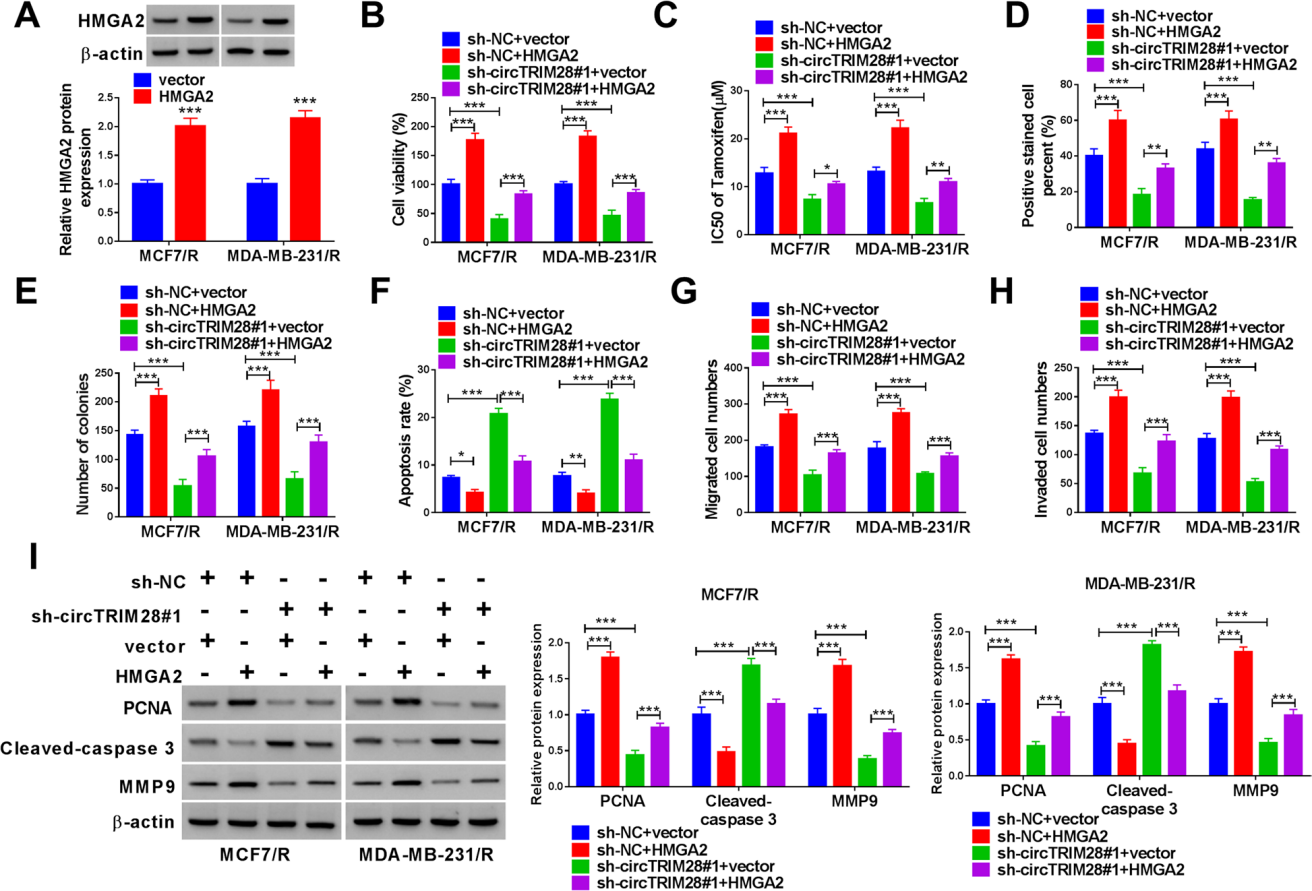
CircTRIM28 expedited BC by regulating HMGA2. **A** The level of HMGA2 was measured. **B** The cell viability, **C** the IC50 of tamoxifen, **D** the cell proliferation, **E** the number of colonies, **F** the rate of apoptosis, **G** and **H** the cell migration and invasion, **I** the protein level of PCNA, Cleaved-caspase 3 and MMP9 were examined. ***P < 0.001

The results indicated that HMGA2 expression was memorably increased in MCF7/R and MDA-MB-231/R cells transfected with HMGA2 compared to the vector group (Fig. 6A). Then, MCF7/R and MDA-MB-231/R cells were transfected with sh-NC + HMGA2 and sh-circTRIM28 + HMGA2, with sh-NC + vector or sh-circTRIM28 + vector as the control. We found the HMGA2 could enhance the cell vitality and IC_50_ value of tamoxifen, and reverted the effect of sh-circTRIM28#1 (Fig. 6B and C). Besides, we established that HMGA2 stimulated cell proliferation and reverted the effect of silencing circTRIM28 (Fig. 6D and E). In addition, the HMGA2 overexpression could inhibit the cell apoptosis, and reverted the effect of sh-circTRIM28#1 (Fig. 6F). Moreover, we observed that HMGA2 overexpression promoted cell invasion and migration and reverted the effect of silencing circTRIM28 (Fig. 6G and H). Afterward, the HMGA2 overexpression could increase the level of PCNA and MMP9 and diminished Cleaved-caspase 3 level and reverted the effect of sh-circTRIM28#1 (Fig. 6I). In conclusion, our findings demonstrated that circTRIM28 deficiency inhibited the progression of BC by regulating HMGA2.

### CircTRIM28 knockdown restricted tumor growth in vivo

**Fig. 7:**
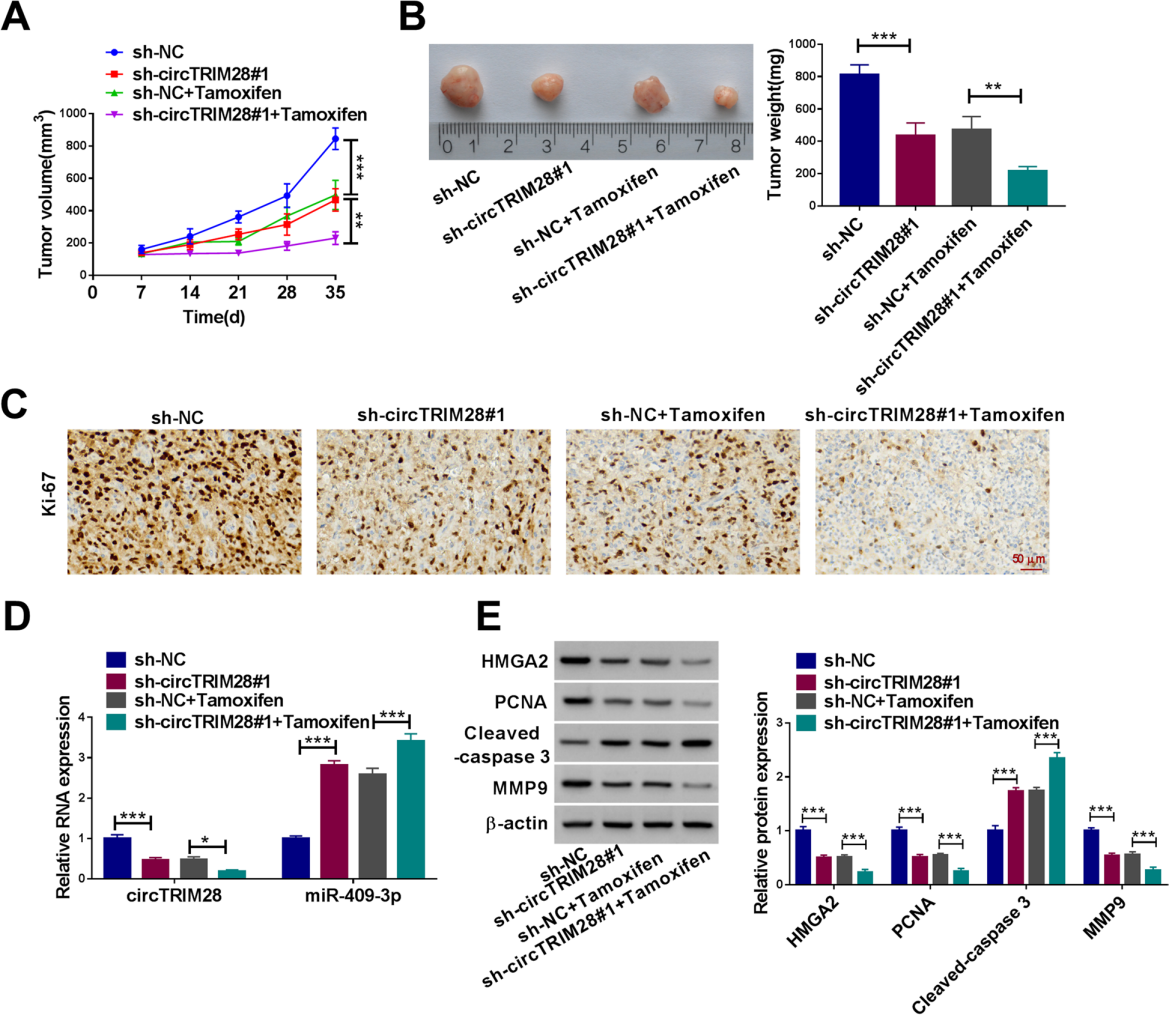
CircTRIM28 deficiency delimited tumor growth. **A** The tumor volume was measured. **B** Tumor weight was detected. **C** Ki-67 content was quantified by IHC. **D** The circTRIM28 and miR-409-3p levels were examined. **E** The protein level of HMGA2, PCNA, Cleaved-caspase 3 and MMP9 were assessed. *P < 0.05, **P < 0.01, ***P < 0.001

MDA-MB-231/R cells were transfected with sh-circTRIM28#1 and sh-circTRIM28#1 + tamoxifen, with sh-NC and sh-NC + tamoxifen as the control. As shown in Fig. 7A and B, tamoxifen treatment repressed tumor volume and weight. Meanwhile, circTRIM28 silencing could also restrain tumor volume and weight. The repression impact is more pronounced when tamoxifen acted synergistically with circTRIM28 downregulation. The Ki67 level was lower after the sh-circTRIM28#1 treatment. And tamoxifen treatment also repressed the expression level of Ki67. When tamoxifen and sh-circTRIM28#1 act synergistically, the inhibitory effect is enhanced (Fig. 7C). This suggests that silencing circTRIM28 enhances the inhibitory effect of tamoxifen on tumor development. The tumor tissues examined the impacts of circTRIM28 knockdown and tamoxifen treatment on circTRIM28, miR-409-3p, HMGA2, PCNA, Cleaved-caspase 3, and MMP9 expression. The results showed that circTRIM28 knockdown or tamoxifen treatment both repressed the expression of circTRIM28, HMGA2, PCNA and MMP9, but amplified the miR-409-3p and Cleaved-caspase 3 levels. The tamoxifen and sh-circTRIM28#1 act synergistically to enhance these effects (Fig. 7D and E). These outcomes signposted that circTRIM28 deficiency repressed tumor growth via miR-409-3p/HMGA2 axis. Besides, circTRIM28 knockdown could improve the drug sensitivity of BC tumors to tamoxifen.

## Discussion

As an anti-estrogen, Tamoxifen has verified its value in the treatment of ER-positive BC. However, current research suggested that resistance to tamoxifen continues to be one of the primary causes of the treatment failure [5, 27]. Previous studies have shown that there are many mechanisms of TAM acquired resistance, among which epigenetic modification is of great importance [28]. Interestingly, this modification could alter gene expression without altering DNA sequence, containing non-coding RNAs. It has been confirmed that circRNAs could perform vital roles in TAM resistance and malignant biological behaviors in BC. For example, a series of circRNAs, such as circ_0025202 and circBMPR2 have been proved to partake in the regulation of tumor development and tamoxifen sensitivity in BC [29, 30]. Furthermore, preceding reporting has been discovered that the under-expression of circCDK1 might repress BC cell development and enhance the sensitivity of tamoxifen [31]. Herein, our research inspected the character of circTRIM28. In the present experiment, circTRIM28, a newly identified circRNA with typical characteristics, was aberrantly upregulated in tamoxifen-resistant BC tissues and cells. Moreover, we found a negative correlation between circTRIM28 high expression and poor prognosis of BC patients, further supporting the potential involvement of circTRIM28 in BC progression. In the functional experiment, our outcomes signposted that the downregulated circTRIM28 enhanced tamoxifen sensitivity and cell apoptosis, whereas inhibited cell proliferation, cell migration, and invasion in BC cells. Apart from that, the chemo-resistance of circTRIM28 was confirmed on BC xenografts in nude mice. These findings indicated that circTRIM28 appears to play a signification role in resistance to tamoxifen in vitro and in vivo.

Currently, one hypothesis is that circRNAs could function as the competing endogenous RNA (ceRNA) to sequester miRNAs away from their target mRNAs [21, 22]. Previous papers suggested that the regulatory networks were also applicable to the study of BC progression and drug resistance [32]. In this study, circTRIM28 was witnessed to increase the speed of BC development by regulating miR-409-3p, which is parallel to preceding outcomes.MiR-409-3p regulated human ovarian cancer, colon cancer, and gastric cancer [33–35]. This evidence disclosed that miR-409-3p took part in human diseases development. Herein, we elucidated that miR-409-3p adjusted the evolution of BC by repressing HMGA2. The upshots confirmed that miR-409-3p could participate in BC.

HMGA2 correlates with the progression of many human cancers. For example, HMGA2 could regulate the cell metastasis of lung cancer [36]. Besides, HMGA2 could modulate the Hippo-YAP signaling pathway to promote BC metastasis [37]. In this work, the expression of HMGA2 was neutralized in BC. Besides, miR-409-3p upregulation repressed BC, but this effect was lessened by HMGA2. These upshots backup the adjusting character of the circTRIM28/miR-409-3p/HMGA2 in BC cells.

## Conclusion

CircTRIM28 and HMGA2 were intensified and miR-409-3p was constrained in BC. Furthermore, our study manifested that circTRIM28 knockdown enhanced tamoxifen sensitivity and cell apoptosis, whereas inhibited cell proliferation, cell migration, and invasion in BC cells via miR-409-3p/HMGA2 axis. This paper manifests a new and feasible treatment idea in BC patients with chemoresistance.

## Data Availability

The analyzed data sets generated during the present study are available from the corresponding author on reasonable request.

## Abbreviations

TAM: Tamoxifen
BC: breast cancer
circRNAs: circular RNAs
circTRIM28: containing 28
miR-409-3p: microRNA-409-3p
HMGA2: high mobility group AT-hook 2
qRT-PCR: quantitative real-time reverse transcription-polymerase chain reaction
HR: Hormone receptor
IHC: Immunohistochemistry
